# Translation, cultural adaptation, and pilot testing of the German cancer worry scale among *BRCA1/2* pathogenic variant carriers in Austria

**DOI:** 10.1186/s13053-025-00316-9

**Published:** 2025-05-19

**Authors:** Anna-Maria Parger, Daniela Muhr, Christian F. Singer, Yen Y. Tan

**Affiliations:** Department of Obstetrics and Gynaecology and Comprehensive Cancer Center, Medical University of Vienna, Waehringer Guertel 18-20, Vienna, 1090 Austria

**Keywords:** BRCA, Cancer worry, Cancer worry scale, Translation, Pilot study

## Abstract

**Background:**

Cancer-related worry can significantly impact psychosocial wellbeing and decision-making, especially among individuals with hereditary cancer risk. Although the Cancer Worry Scale is a commonly used instrument, no culturally adapted version exists for German speaking populations. This study aimed to translate, culturally adapt and pilot-test a German version of the 8-item Cancer Worry Scale in individuals carrying *BRCA1* or *BRCA2* pathogenic variants in Austria.

**Methods:**

The scale was translated using a forward and backward translation process, and reviewed by an expert panel. Participants were recruited from a familial cancer clinic and completed the translated scale along with demographic questions. Participants provided feedback on item clarity and comprehension, which informed minor revisions. The final version was then pilot-tested with a small sample of *BRCA1/2 *carriers.

**Results:**

Thirty-five individuals with *BRCA1/2* pathogenic variants completed the scale. Most participants found the scale understandable, though eight reported difficulties with certain items. Based on this feedback, four items were revised to improve clarity. Descriptive analysis indicated similar worry patterns to those observed in international studies. Women who had not undergone risk-reducing surgery reported higher cancer worry, while male participants expressed elevated concern primarily for the health of their family members.

**Conclusion:**

This pilot study presents the first pilot-tested German version of the 8-item Cancer Worry Scale. While initial results support its feasibility and comprehension, further research is needed to validate the psychometric properties of the instrument in larger German-speaking populations.

**Supplementary Information:**

The online version contains supplementary material available at 10.1186/s13053-025-00316-9.

## Introduction

Breast cancer (BC) is the most common cancer affecting 1 in 8 women in their lifetime [1]. While most breast cancer cases are sporadic, about 5–10% are hereditary [2], often associated with pathogenic variants in the *BRCA1* or *BRCA2* (*BRCA1/2* hereafter) genes. Carriers of *BRCA1* germline pathogenic variants have a significantly high lifetime risk of breast cancer – up to 72%, whereas *BRCA2* carriers have a slightly lower but still a substantial risk of around 69% [3]. Ovarian cancer risks also differ, with *BRCA1* carriers facing up to 44% compared to 17% for *BRCA2* carriers. Although the incidence of BC is rare in men, carriers of the *BRCA1/2* pathogenic variants have an increased risk of up to 10% (versus 0.1% in the general male population) and are also at higher risk for prostate cancer (15% vs. 6% in the general population). Both male and female *BRCA1/2* carriers also have a 5% increased risk for pancreatic, bladder or gastrointestinal cancers [4, 5].

Receiving a positive *BRCA1/2* test result can significantly impact psychological well-being, as individuals face complex decisions regarding cancer risk management [6–12]. Some degree of worry is considered normal, but persistent high cancer worry can cause social and emotional dysfunction, impair quality of life, and hinder engagement with supportive care services [12]. Timely identification and management of elevated cancer worry are therefore critical components of comprehensive care for *BRCA1/2* carriers.

The Cancer Worry Scale (CWS), originally developed by Lerman et al. is a 4-item scale to assess worry about cancer among breast cancer survivors [13]. Later, Douma et al. expanded the scale to 8-items, providing a more comprehensive assessment capturing emotional and functional consequences of worry, making it more suitable for broader application in both survivor and high-risk cohorts [14]. Over time, the CWS has been adapted, validated and extensively used in various context among cancer survivors [14–17] and in populations with hereditary cancer risk, such as *BRCA1/2* carriers [10, 18]. In German-speaking populations, no officially translated or culturally adapted version of the full 8-item CWS exist. A prior attempt at translating the 4-item version was conducted as part of a student thesis; however, this work lacked methodological rigor and was limited to the shortened scale [19]. Given the growing need for validated, culturally sensitive measures of cancer worry among *BRCA1/2* carriers, this study aimed to translate, culturally adapt, and pilot-test the German version of the full 8-item CWS in a sample of *BRCA1/2* pathogenic variant carriers in Austria. The primary objective was to assess initial feasibility and comprehension of the translated scale, with formal psychometric validation reserved for a larger future study. By establishing a German version of the CWS, the study hopes to facilitate the accurate assessment of cancer worry, ultimately contributing to more targeted psychological support and intervention strategies.

## Materials and methods

### Study design

This study employed a two-phase design to translate, culturally adapt, and pilot test the 8-item CWS for German-speaking individuals in Austria with *BRCA1/2* pathogenic variants. As a pilot study, the sample size was pragmatically determined based on the number of *BRCA1/2*-pathogenic carriers identified through the clinical genetics registry, ATHENA, at our center.

### Phase 1: translation and cultural adaptation

Using the original English version with permission, we translated the eight items into German according to international guidelines [20, 21], using forward and backward translation. The English version of the CWS was translated into German by a bilingual interpreter. The preliminary German version was then reviewed by a committee comprising two medical doctors, a breast care nurse, and a psychologist, who refined the translation for clarity and cultural relevance. Following this review, a second independent bilingual translator performed back-translation into English to check for consistency with the original text. Any discrepancies resulting from the translation were discussed and resolved by the committee to produce a finalized German version of the CWS.

### Phase 2: pilot testing

The final German version of the CWS was piloted with a small sample of female and male *BRCA1/2* pathogenic variant carriers identified through genetic counselling and testing at our center. The aim was to assess initial feasibility – defined as ease of administration and participants’ willingness to complete the questionnaire – and comprehension, based on informal feedback on item clarity. No formal psychometric analyses (e.g. Cronbach’s alpha) were conducted at this stage, as the primary goal was to refine the instrument for future large-scale validation studies.

### Eligible participants

We included adults (≥18 years) who were tested between 2015 and 2020, carried a *BRCA1/2* germline pathogenic variant, had no active cancer diagnosis and were able to provide informed consent for both testing and research participation. In line with recommendations for pilot studies [22, 23], we mailed invitations to 63 eligible individuals by post (with an expected response rate of about 50%). The invitation package included a study information sheet, consent form and the translated CWS.

### Data collection

Participants were asked to complete the German CWS along with a demographic questionnaire, collecting information on education, marital status, employment status, income, and health-related behaviors such as physical activity and if any risk-reducing surgery had been undertaken. Follow-up calls were made two and four weeks after the initial invitation to encourage responses from non-responders. Participant were also asked to provide feedback on clarity and comprehensibility of the translated CWS items for refinement purposes. The responses were then collated and assessed with another independent, professional, bilingual translator (U.C.). The wording of the sentences was revised, while maintaining the context of the original items. The discrepancies were discussed and reviewed by the committee until a consensus was reached on all items. This study has been approved by the Ethics Commission of Medical University of Vienna (EK1869/2020). All participants provided informed consent.

### Data analyses

The purpose of our pilot study is to show feasibility and comprehension rather than confirm specific hypothesis or making definite conclusion. As such, the primary analyses were descriptive and exploratory. The comparisons between *BRCA1* and *BRCA2* carriers were informed by established clinical differences in cancer risk and management, as well as prior evidence suggesting differing patterns of cancer worry [18, 24]. No formal power or sample size calculation was performed, as is typical for pilot studies. Both female and male participants were included into the study and gender information was collected based on participants’ self-identification. No separate question regarding sex assigned at birth was included. Surgery variables like salpingo-oophorectomy were relevant only for female participants; male participants were excluded from these analyses.

CWS scores were summarized both as mean total scores and as categorical proportions for individual items. For each item, there are 4 possible response options, ranging from 1 (low worry) to 4 (highest worry), resulting in a total possible score between 8 and 32. A total score of 12 or higher indicates high level of worry, whereas a score below 12 indicates low level of worry among healthy high-risk individuals (such as those in our cohort). Single item responses were collapsed into two categories - “never/sometimes” versus “often/always” - to enable meaningful item-level analysis. While individual item responses were analyzed categorically to improve interpretability, the full CWS score was treated as a continuous variable to preserve variability and statistical sensitivity, in line with standard practice for scale-based instruments [15, 18, 25, 26].

Descriptive statistics such as absolute numbers, percentages, mean, standard deviation, median, 95% confidence interval (CI) and range were calculated for demographic variables. Group comparisons (*BRCA1* versus *BRCA2*) were made using t-tests or ANOVA for normally distributed data, and Mann-Whitney-U or Kruskal-Wallis tests for non-normally distributed data. Chi-square tests were performed to assess categorical outcomes. Two-sided p-values of < 0.05 were considered as statistically significant. Responses with missing values were excluded from relevant analyses, and no adjustments for multiple testing were performed. All statistical analysis were performed using SPSS v27.0 for Mac OS.

## Results

### Participant characteristics

Of the 63 invited individuals, 50 (79%) were women and 13 (21%) were men. Thirty-five individuals completed the questionnaire, giving a response rate of 56%. Only eight of 35 participants provided written feedback, identifying specific items that were unclear or redundant. The most frequently noted issue was the similarity between Items 4 and 5, which were reported as very similar by four participants. Items 1 and 4 were also flagged by two participants for overlapping content. In addition, Items 1,3,6,7 and 8 were described as containing complicated sentence structures that affected understandability. Two participants suggested to replace the term “rarely” with “never” in Items 5 and 7 to improve phrasing. Based on this feedback, Items 1,4,7, and 8 were revised to enhance clarity, reduce redundancy and improve linguistic precision.

The average age of the respondents was 46 years (range 26–64 years). All were native German speakers. Most participants were married or have a life partner (83%) and had completed compulsory school (54%). Additionally, 57% had undergone some risk-reducing surgery, such as mastectomy, salpingo-oophorectomy, salpingectomy or both, mastectomy and salpingo-oophorectomy. Overall patients’ characteristics by mutation type is shown in Table 1.

**Table 1:** Patients’ characteristics by *BRCA* status (total = 35)

Characteristics	*BRCA1* *N* (%)	*BRCA2* *N* (%)	*p*-value
Total	28 (80)	7 (20)	
Age in years (range)	40 (26–68)	51 (30–64)	0.028
Gender^a^			
Male	4 (14)	1 (14)	0.999
Female	24 (86)	6 (86)	
Marital status			
Married or life partner	22 (79)	7 (100)	0.311
Other^b^	6 (21)	0	
Education			
Compulsory school^c^	15 (54)	4 (57)	0.811
High school diploma	6 (21)	2 (29)	
University degree	7 (25)	1 (14)	
Employment status			
Full-time	14 (50)	2 (29)	0.173
Part-time	8 (29)	1 (14)	
Other^d^	6 (21)	4 (57)	
Income status			
Low income	8 (29)	3 (43)	
Average/high income^e^	20 (71)	4 (57)	0.652
Sports			
Never	8 (30)	3 (43)	
At least two times a week	10 (37)	2 (28,5)	
At least 3 times a week	9 (33)	2 (28,5)	0.798
Missing	1		
Risk reducing surgery			
No	12 (44)	3 (43)	
Mastectomy	7 (26)	1 (14)	
Salpingo-oophorectomy	3 (11)	2 (29)	
Salpingectomy	1 (4)	1 (14)	
Mastectomy and salpingo-oophorectomy	4 (15)	0	0.467
Missing	1		

^a^ self-reported gender

^b^ single, divorced or separated, widowed

^c^ In Austria, mandatory school years are up to age 15

^d^ in training, unemployed, maternal leave, sick leave, retirement

^e^ more than 18.000 Euros net income per year

### Cancer worry levels

Of 35 respondents who completed the CWS, 27 (77%) reported high levels of cancer worry (mean = 14, SD = 3.5), with no statistically significant difference in worry observed between *BRCA1* and *BRCA2* carriers (81% vs. 71%, respectively; *p* = 0.615). While most participants frequently thought about their risk of developing cancer, few reported that these thoughts affected their mood (11%) or daily activities (0%).

*BRCA2* carriers were significantly more concerned about needing additional surgery than *BRCA1* carriers (57% vs. 7%, respectively; *p* = 0.001). Although insignificant, *BRCA2* carriers were more concerned about the possibility of getting cancer one day (*p* = 0.66) and about developing cancer (*p* = 0.27) but they worry less about their family members developing cancer (*p* = 0.28) (Table 2). However, there was no observed interference from cancer worry with participants’ daily activities.

**Table 2:** 8-items Cancer worry scale by BRCA status [14, 15]

Items of the Cancer Worry Scale referring to the last 6 months		*BRCA1* *N* (%) 28 (80%)		*BRCA2* *N* (%) 7 (20%)	
		Never/ sometimes	Often/ always	Never/ sometimes	Often/ always
1	How often have you thought about your chances of getting cancer?	24 (86)	4(14)	5 (71)	2 (29)
2	Have these thoughts affected your mood?	25 (88)	3 (12)	6 (86)	1 (14)
3	Have these thoughts interfered with your ability to do daily activities?	28 (100)	0	7 (100)	0
4	How concerned are you about the possibility of getting cancer one day?	24(86)	4 (14)	4(57)	3 (43)
5	How often do you worry about developing cancer? *	24 (89)	3 (11)	5 (71)	2 (29)
6	How much of a problem is this worry?	27 (96)	1 (4)	4 (57)	3 (43)
7	How often do you worry about the chance of family members developing cancer?	18 (64)	10 (36)	6 (86)	1 (14)
8	How concerned are you about the possibility that you will ever need surgery (again)?	26 (93)	2 (7)	3 (43)	4 (57)

* one participant did not respond to this question

### Factors influencing cancer worry

**Fig. 1:**
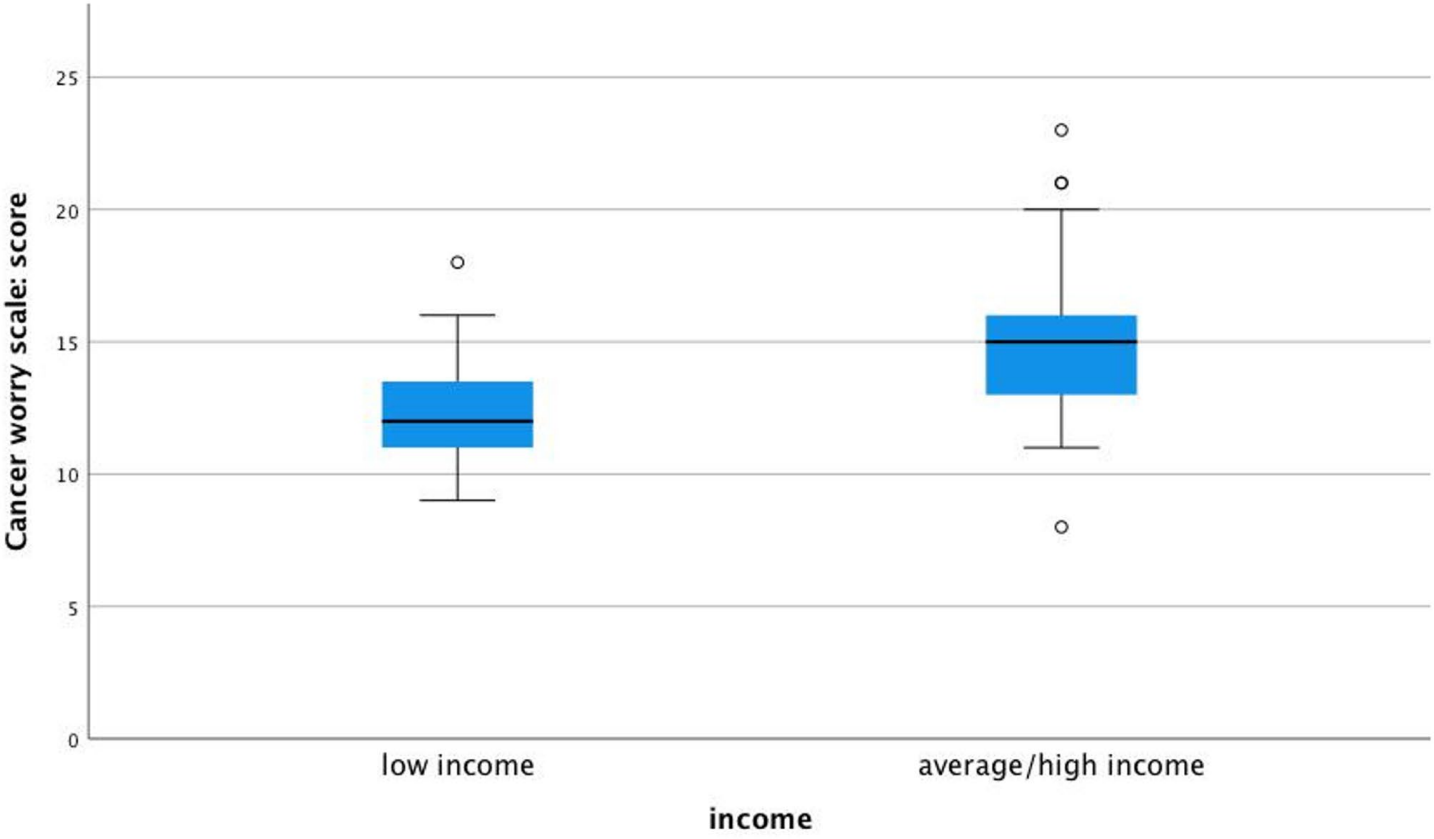
Significantly higher cancer worry scores are reported by participants with average/high income versus low income, *p* = 0.024

**Fig. 2:**
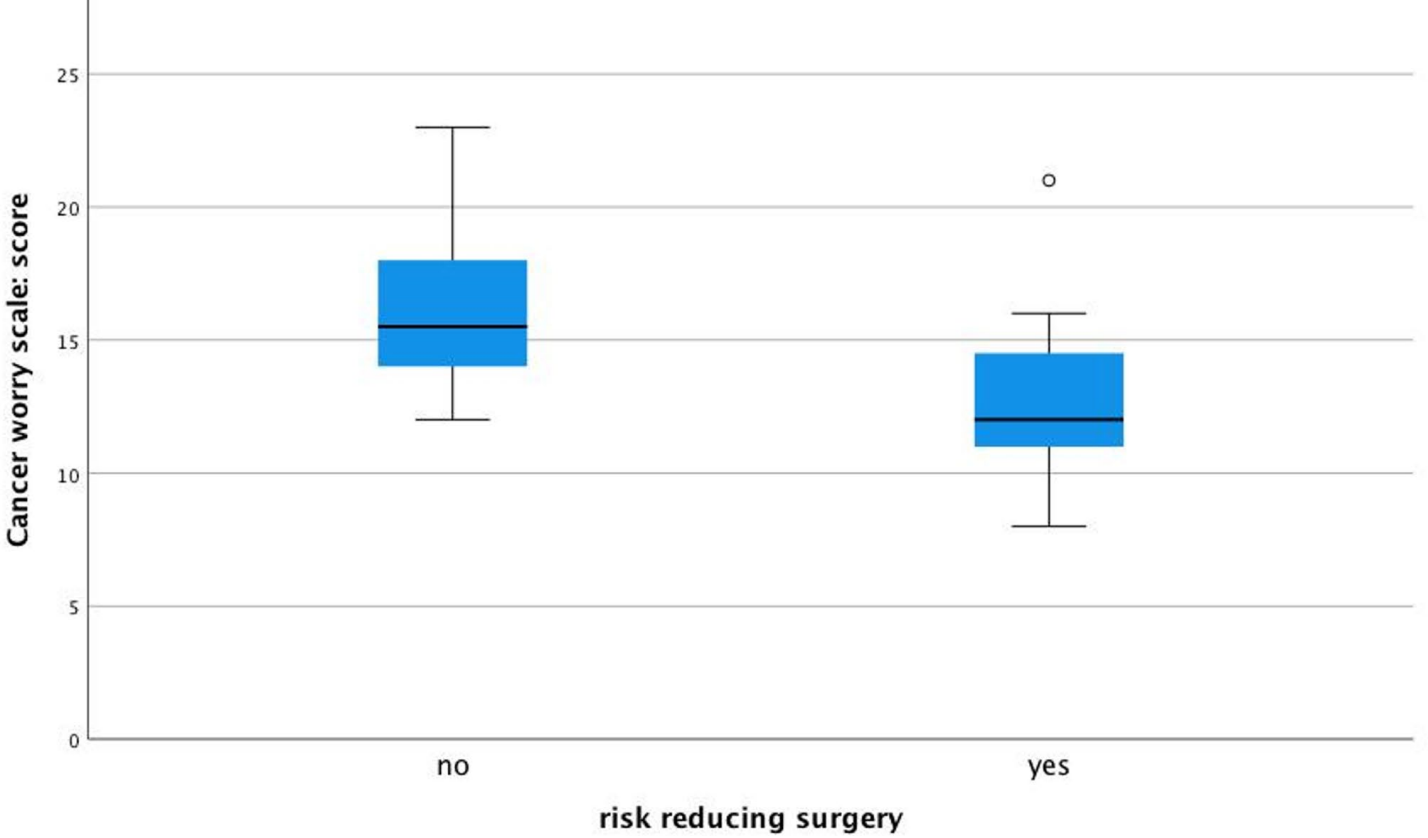
Significantly lower cancer worry scores are reported by those who underwent risk-reducing surgery than those who did not, *p* = 0.014

Participants with average or high incomes reported significantly higher cancer worries than those with lower incomes (91% vs. 54%, respectively; *p* = 0.024; Fig. 1). Those who had undergone risk-reducing surgery (mastectomy and/or salpingo-oophorectomy) reported significantly lower levels of cancer worry compared to those who had not (*p* = 0.014, Fig. 2). This association was observed across multiple CWS items but should be interpreted with caution, as potential confounding variables were not adjusted for due to small sample size. No significant differences in cancer worry were observed based on marital status (*p* = 0.306), education level (*p* = 0.875), employment status (*p* = 0.064), or physical activity level (*p* = 0.715).

### Gender differences in cancer worry

Although no significant gender-based difference was found in overall worry levels, all male participants reported high cancer worry compared to female participants (100% vs. 73%, respectively). Although not significant, male participants expressed greater concerns about family members developing cancer compared to female participants (60% vs. 24%, respectively, *p* = 0.43).

## Discussion

This pilot study focused on the translation, culturally adaptation, and initial pilot application of the German 8-item CWS among individuals carrying *BRCA1* or *BRCA2* germline pathogenic variants. The findings provide preliminary insights into the feasibility and comprehension of the translated instrument in a German-speaking population. Participants generally found the scale understandable, although minor wording modifications were made to improve clarity based on participant feedback.

In our study, we acknowledge the prior work of Vodermaier [19], who conducted a German translation of the 4-item CWS as part of a student thesis exploring the psychological consequences of prophylactic surgery among individuals at general risk for breast and ovarian cancer. While this earlier work contributed to initial efforts in assessing cancer worry in German-speaking populations, the methodology lacked comprehensive detail, mentioning only a back-translation process without further elaboration. Moreover, it focused solely on the abbreviated 4-item version of the CWS, limiting its scope in capturing the full range of cancer-related anxiety. Our study addresses these gaps by providing a rigorous adaptation of the complete 8-item CWS into German. This extended version allows for a more comprehensive assessment of cancer worry, particularly tailored to *BRCA1/2* carriers — a group with distinct genetic risks and psychological needs. This extended version captures a broader spectrum of concerns, offering greater sensitivity in identifying and understanding cancer-related anxiety, particularly among *BRCA1/2* carriers, who are a special population due to their unique genetic predisposition and specific psychological needs.

Our results showed no significant difference in cancer worry levels between *BRCA1* and *BRCA2* carriers. However, individuals with *BRCA2* variants expressed greater concerns about future surgeries, which may relate to a lower uptake of risk-reducing procedures in this subgroup within our sample. This finding aligns with previous reports that show women who undergo preventive surgeries tend to report lower levels of cancer worry, possibly due to a sense of increased control and reduced perceived risk [7, 18, 27–29]. International studies have shown that the uptake of risk-reducing surgeries among *BRCA1/2* carriers varies widely, influenced by factors such as age, country of residence, perceived cancer risk, cultural norms, and access to genetic counseling. For example, uptake rates for risk-reducing mastectomy range from approximately 30–60% and for salpingo-oophorectomy from 50 to 85%, depending on the setting [30–32]. These differences highlight the importance of considering cross-cultural factors when interpreting cancer worry and decision-making around surgery. In addition to reducing perceived risk, risk-reducing surgeries have also been associated with improvements in quality of life [7, 33, 34].

Interestingly, our study showed that average or higher income levels were associated with increased cancer worry. Although we were not able to adjust for confounding variables, e.g. education level, due to small sample size, this finding is consistent with other studies where socioeconomic status is associated with heightened health anxiety. For instance, a study of 1773 healthy women reported individuals with higher income have elevated cancer worry and were found to be more likely to undergo screening for BC [35]. Similarly, a population-based study reported that being employed was correlated with higher cancer worry about BC recurrence [36]. This may be due to the increased perceived importance of career and lifestyle consequences, which could, in turn, result in a decreased family income. Like other studies, our study suggests that preventive interventions may alleviate worry by reducing perceived risk, thus highlighting the psychological benefit of such procedures.

Gender differences, although not statistically significant, highlight an area for further exploration. Fewer male participated compared to women, but they reported a higher level of concern about the possibility of family members developing cancer. Fewer male participants were expected since breast cancer is rare and only occurring in < 1% of all BC diagnosis [5]. However, our findings align with other studies showing that men with *BRCA1/2* variants, despite being less frequently affected by breast cancer themselves, experience considerable worry for their female family members. For instance, a study with men aged 25–60 years, who are brothers of women with familial breast cancer, showed that the men were concerned that their daughters might develop BC [37]. In another study, men were also reported to be less included in family discussions about BC and felt uncomfortable with being at risk for a “women’s disease” [38]. This might lead to unspoken worries and concerns in male *BRCA1/2* carriers leading to poorer quality of life and unbalanced supportive care needs for this subset of patients, further enlarging the health inequality gap between gender. These findings therefore highlight the importance of including men in genetic counselling and support interventions to address their concerns and improve family-centered care.

There are limitations to our study. First, this was a small pilot study designed to assess initial feasibility and comprehension, without conducting formal psychometric validation. Measures such as internal consistency (e.g. Cronbach’s alpha), test-retest reliability, and construct validity were not assessed and will be the focus of future larger-scale studies. As such, findings from our study need to be interpreted with caution and results should be treated as hypothesis-generating. Second, while participant feedback guided minor modifications to item wording, the evaluation of item clarity was informal and based on participant comments rather than structured cognitive interviewing. Third, due to the sample size, we were unable to perform multivariate analyses to adjust for potential confounding factors, such as age or educational level. Finally, gender was self-reported, and no separate assessment of sex assigned at birth was conducted, which may limit the interpretation of risk-reducing surgery variables.

### Clinical implications

The German version of the Cancer Worry Scale allows for the evaluation of patient’s needs and concerns, enabling clinicians to identify individuals experiencing elevated cancer worry and offer appropriate psychological support.

## Conclusion

In summary, this study represents a first step toward making the CWS accessible to German-speaking high-risk population. Through careful translation, cultural adaptation and pilot application among *BRCA1/2* pathogenic variant carriers, we have laid the groundwork for future validation. As cancer worry plays a critical role in decision-making and psychosocial outcomes, a reliable and culturally appropriate tool is essential. Further research with larger sample size is now warranted to confirm the psychometric properties of the German CWS and to support its broader implementation in clinical and research settings.

## Supplementary Information

Supplementary Material 1.

## Data Availability

No datasets were generated or analysed during the current study.
