# Supplementary material for: Translation, cultural adaptation, and pilot testing of the German cancer worry scale among BRCA1/2 pathogenic variant carriers in Austria
Source: Hered Cancer Clin Pract. 2025 May 19;23:17. doi: 10.1186/s13053-025-00316-9 (PMC12087244; doi:10.1186/s13053-025-00316-9)
Supplement: Supplementary file 1 — Supplementary Material 1. [file 13053_2025_316_MOESM1_ESM.docx]

**Supplementary Tables**

*Supplementary Table 1: The 8-item Cancer Worry Scale, English version [*[*1*](#_ENREF_1)*,* [*2*](#_ENREF_2)*]*

During the past six months:

1. How often have you thought about your chances of getting cancer (again)?

| **almost never** | **sometimes** | **often** | **almost always** |
| --- | --- | --- | --- |
| 1 | 2 | 3 | 4 |

2. Have these thoughts affected your mood?

| **Almost never** | **Sometimes** | **Often** | **Almost always** |
| --- | --- | --- | --- |
| 1 | 2 | 3 | 4 |

3. Have these thoughts interfered with your ability to do daily activities?

| **Almost never** | **Sometimes** | **Often** | **Almost always** |
| --- | --- | --- | --- |
| 1 | 2 | 3 | 4 |

4. How concerned are you about the possibility of getting cancer (again) one day?

| **Not at all** | **A little** | **Quite a bit** | **Very much** |
| --- | --- | --- | --- |
| 1 | 2 | 3 | 4 |

5. How often do you worry about developing cancer (again)?

| **Almost never** | **Sometimes** | **Often** | **Almost always** |
| --- | --- | --- | --- |
| 1 | 2 | 3 | 4 |

6. How much of a problem is this worry?

| **Not at all** | **A little** | **Quite a bit** | **Very much** |
| --- | --- | --- | --- |
| 1 | 2 | 3 | 4 |

7. How often do you worry about the chance of family members developing cancer?

| **Almost never** | **Sometimes** | **Often** | **Almost always** |
| --- | --- | --- | --- |
| 1 | 2 | 3 | 4 |

8. How concerned are you about the possibility that you will ever need surgery (again)?

| **Not at all** | **A little** | **Quite a bit** | **Very much** |
| --- | --- | --- | --- |
| 1 | 2 | 3 | 4 |

1. Douma, K.F., et al., *Psychological distress and use of psychosocial support in familial adenomatous polyposis.* Psychooncology, 2010. **19**(3): p. 289-98.

2. Custers, J.A., et al., *The Cancer Worry Scale: detecting fear of recurrence in breast cancer survivors.* Cancer Nurs, 2014. **37**(1): p. E44-50.

*Supplementary Table 2: The 8-item Cancer Worry Scale – German, pre-pilot version*

In den **letzten 6 Monaten:**

1.. Wie oft haben Sie an die Wahrscheinlichkeit gedacht, (wieder) an Krebs zu erkranken?

| **selten** | **manchmal** | **oft** | **fast immer** |
| --- | --- | --- | --- |
| 1 | 2 | 3 | 4 |

2. Haben diese Gedanken Ihre Stimmung beeinflusst?

| **selten** | **manchmal** | **oft** | **fast immer** |
| --- | --- | --- | --- |
| 1 | 2 | 3 | 4 |

3. Haben diese Gedanken Ihre Fähigkeit beeinflusst, alltägliche Aktivitäten zu verrichten?

| **selten** | **manchmal** | **oft** | **fast immer** |
| --- | --- | --- | --- |
| 1 | 2 | 3 | 4 |

4. Wie besorgt sind Sie über die Möglichkeit, eines Tages (wieder) an Krebs zu erkranken?

| **gar nicht** | **ein wenig** | **ziemlich viel** | **sehr viel** |
| --- | --- | --- | --- |
| 1 | 2 | 3 | 4 |

5. Wie oft sorgen Sie sich, (wieder) an Krebs zu erkranken?

| **selten** | **manchmal** | **oft** | **fast immer** |
| --- | --- | --- | --- |
| 1 | 2 | 3 | 4 |

6. Wie sehr belasten Sie diese Sorgen?

| **gar nicht** | **ein wenig** | **ziemlich viel** | **sehr viel** |
| --- | --- | --- | --- |
| 1 | 2 | 3 | 4 |

7. Wie oft sorgen Sie sich über die Wahrscheinlichkeit, dass Familienmitglieder an Krebs erkranken?

| **selten** | **manchmal** | **oft** | **fast immer** |
| --- | --- | --- | --- |
| 1 | 2 | 3 | 4 |

8. Wie besorgt sind Sie über die Wahrscheinlichkeit, dass Sie jemals (wieder) operiert werden müssen?

| **gar nicht** | **ein wenig** | **ziemlich viel** | **sehr viel** |
| --- | --- | --- | --- |
| 1 | 2 | 3 | 4 |

*Supplementary Table 3: The final 8-item Cancer Worry Scale – German, post-pilot version*

In den **letzten 6 Monaten:**

1.. Wie oft haben Sie sich Gedanken darüber gemacht, ob Sie (wieder) an Krebs erkranken könnten?

| **fast nie** | **manchmal** | **oft** | **fast immer** |
| --- | --- | --- | --- |
| 1 | 2 | 3 | 4 |

2. Haben diese Gedanken Ihre Stimmung beeinflusst?

| **fast nie** | **manchmal** | **oft** | **fast immer** |
| --- | --- | --- | --- |
| 1 | 2 | 3 | 4 |

3. Haben diese Gedanken Ihre Fähigkeit, Alltagsaktivitäten nachzugehen, beeinträchtigt?

| **fast nie** | **manchmal** | **oft** | **fast immer** |
| --- | --- | --- | --- |
| 1 | 2 | 3 | 4 |

4. Machen Sie sich Sorgen, dass Sie eines Tages (wieder) an Krebs erkranken könnten?

| **überhaupt nicht** | **ein wenig** | **ziemlich viel** | **sehr viel** |
| --- | --- | --- | --- |
| 1 | 2 | 3 | 4 |

5. Wie oft sorgen Sie sich, (wieder) an Krebs zu erkranken?

| **fast nie** | **manchmal** | **oft** | **fast immer** |
| --- | --- | --- | --- |
| 1 | 2 | 3 | 4 |

6. Wie sehr belasten Sie diese Sorgen?

| **überhaupt nicht** | **ein wenig** | **ziemlich viel** | **sehr viel** |
| --- | --- | --- | --- |
| 1 | 2 | 3 | 4 |

7. Wie oft sorgen Sie sich, dass Familienmitglieder an Krebs erkranken könnten?

| **fast nie** | **manchmal** | **oft** | **fast immer** |
| --- | --- | --- | --- |
| 1 | 2 | 3 | 4 |

8. Wie sehr beschäftigt Sie der Gedanke, dass Sie irgendwann (wieder) operiert werden müssen?

| **überhaupt nicht** | **ein wenig** | **ziemlich viel** | **sehr viel** |
| --- | --- | --- | --- |
| 1 | 2 | 3 | 4 |

Supplementary Table 4: Items of the Cancer Worry Scale (CWS) in relation to risk-reducing surgery

|  | Items of the CWS referring to the past 6 months | **Risk reducing surgery^1^** | | | |
| --- | --- | --- | --- | --- | --- |
|  |  | **Yes**  n (%) | | **No**  n (%) | |
|  |  | Never/  sometimes | Often/ always | Never/  sometimes | Often/ always |
| 1 | How often have you thought about your chances of getting cancer? | 18 (95) | 1 (5) | 11 (73) | 4 (27) |
| 2 | Have these thoughts affected your mood? | 17 (89) | 2 (11) | 14 (93) | 1 (7) |
| 3 | Have these thoughts interfered with your ability to do daily activities? | 19 (100) | 0 | 15 (100) | 0 |
| 4 | How concerned are you about the possibility of getting cancer one day? | 18 (95) | 1 (5) | 10 (67) | 5 (33) |
| 5 | How often do you worry about developing cancer? * | 11 (56) | 8 (42) | 3 (20) | 11 (80) |
| 6 | How much of a problem is this worry? | 18 (95) | 1 (5) | 12 (80) | 3 (20) |
| 7 | How often do you worry about the chance of family members developing cancer? | 15 (79) | 4 (21) | 9 (60) | 6 (40) |
| 8 | How concerned are you about the possibility that you will ever need surgery (again)? | 17 (89) | 2 (11) | 11 (73) | 4 (27) |
| ^1^ Includes risk reducing mastectomy, risk reducing salpingo-oophorectomy, or both; one participant did not answer if risk reducing surgery was performed  ^2^ one participant did not respond to this question | | | | | |
